# Population genetic structure of *Bellamya aeruginosa* (Mollusca: Gastropoda: Viviparidae) in China: weak divergence across large geographic distances

**DOI:** 10.1002/ece3.1673

**Published:** 2015-10-13

**Authors:** Qian H. Gu, Martin Husemann, Baoqing Ding, Zhi Luo, Bang X. Xiong

**Affiliations:** ^1^ College of FisheriesHuazhong Agricultural UniversityWuhanChina; ^2^ General ZoologyInstitute of BiologyMartin‐Luther University Halle‐WittenbergD‐06120Halle (Saale)Germany; ^3^ Department of Ecology and Evolutionary BiologyUniversity of ConnecticutStorrsCT 06269‐3043

**Keywords:** Cytochrome oxidase I, differentiation, freshwater snail, microsatellite, Yangtze River

## Abstract

*Bellamya aeruginosa* is a widely distributed Chinese freshwater snail that is heavily harvested, and its natural habitats are under severe threat due to fragmentation and loss. We were interested whether the large geographic distances between populations and habitat fragmentation have led to population differentiation and reduced genetic diversity in the species. To estimate the genetic diversity and population structure of *B. aeruginosa*, 277 individuals from 12 populations throughout its distribution range across China were sampled: two populations were sampled from the Yellow River system, eight populations from the Yangtze River system, and two populations from isolated plateau lakes. We used seven microsatellite loci and mitochondrial cytochrome oxidase I sequences to estimate population genetic parameters and test for demographic fluctuations. Our results showed that (1) the genetic diversity of *B. aeruginosa* was high for both markers in most of the studied populations and effective population sizes appear to be large, (2) only very low and mostly nonsignificant levels of genetic differentiation existed among the 12 populations, gene flow was generally high, and (3) relatively weak geographic structure was detected despite large geographic distances between populations. Further, no isolation by linear or stream distance was found among populations within the Yangtze River system and no signs of population bottlenecks were detected. Gene flow occurred even between far distant populations, possibly as a result of passive dispersal during flooding events, zoochoric dispersal, and/or anthropogenic translocations explaining the lack of stronger differentiation across large geographic distances. The high genetic diversity of *B. aeruginosa* and the weak population differentiation are likely the results of strong gene flow facilitated by passive dispersal and large population sizes suggesting that the species currently is not of conservation concern.

## Introduction

The level of genetic diversity in natural populations is determined by the interplay of mutation, migration, hybridization, drift, and selection (Harrison 1991; Vellend and Geber 2005). The relative role that each force plays depends on life‐history traits, the mating system, and the dispersal ability of a species. Extrinsic factors such as the landscape matrix, the geographic history, and anthropogenic actions can further affect genetic diversity of natural populations (Husemann et al. 2012; Fernández‐García et al. 2014; Eberhart‐Phillips et al. 2015). The landscape configuration can influence dispersal, which in turn affects the population genetic structure. For example, mountainous landscapes and anthropogenically fragmented systems may increase the genetic divergence among natural populations by limiting seasonal movements (Epps et al. 2005; Roffler et al. 2014). The geographic history of a landscape, in turn, has influenced the demographic history of the local populations, which is an important factor shaping the current genetic composition of a species. Demographic bottlenecks and founder effects in populations at the leading edge, for example, can lead to reduced genetic diversity (Ray et al.^2015^). Hence, the population structure that can be observed today is the result of a complex interplay of current and past processes which are difficult to disentangle. In order to understand the contributions of both historical and contemporary factors, the use of markers with different evolutionary speeds can be an important resource.

Freshwater gastropods represent interesting models to study the effects of extrinsic factors on the population genetic structure due to their primarily sessile lifestyle (Nekola 2012). Many snail species show strong genetic differentiation between populations, even across small geographic distances (Hurtrez‐Boussès et al. 2010; Tian‐Bi et al. 2013). However, freshwater snails often occur in high abundances (Chaine et al. 2012) potentially reducing the amount of genetic drift that populations experience counteracting genetic differentiation between populations (Tibbets and Dowling 1996). Further, passive dispersal via drifting of larvae or adults may reduce genetic differentiation, at least for riverine species within river catchments. Long‐distance dispersal, however, is generally only possible if snails or their larvae are transported passively, either by animals (zoochory) or accidentally or on purpose by humans (anthropogenic translocation) (Gittenberger et al. 2006; Gittenberger 2012).

Despite multiple modes of passive dispersal, strong population divergence is often observed between local snail populations (Sinclair‐Winters 2014), either as a result of natural geographic isolation or anthropogenic fragmentation. The genetic divergence is often accompanied by elevated effects of drift (González‐Astorga and Núñez‐Farfán 2001) and a reduction in genetic diversity and effective population size (*N*_e_) (Zuberogoitia et al. 2013). Such reductions may have negative effects on the adaptive potential of populations (Reed and Frankham 2003; Willi et al. 2006). Therefore, estimating genetic diversity and differentiation can provide important insights into the threat status of a species (Toro and Caballero 2005); further, analyses of genetic structure and demographic analyses using multiple genetic markers with different evolutionary speeds may help to understand the factors that are and have been most relevant in shaping a population's genetic structure (Waples and Gaggiotti 2006; Roberts et al. 2013).

Here, we investigate the population structure and distribution of genetic diversity in the Chinese freshwater snail *Bellamya aeruginosa*. The species belongs to a species‐rich genus of primary sessile freshwater snails, which is widely distributed across South‐East Asia, India and Africa (Schultheiß et al. 2011; Van Bocxlaer and Hunt 2013). *Bellamya aeruginosa* is a relatively common species in China and has a wide distribution across the Yangtze and Yellow river systems. In particular, the large number of great lakes in the Yangtze River system, specifically in the Jianghan plain and Dongting Lake Plain, provides an outstanding environment for *B. aeruginosa*. However, due to intensive exploitation (Ozawa et al. 2003; Song et al. 2007), the species may be of conservation concern; yet, formal evaluations of the status of the species have not been performed in this area. Further, the species may be affected by the strong fragmentation of Chinese river systems by damming in the last century (Nilsson et al. 2005), especially in Yangtze River system, potentially further reducing its population size and genetic diversity (Ricciardi and Rasmussen 1999; Roberts et al. 2013).

In this study, we sampled 10 populations of *B. aeruginosa* across a network of large lakes in China that are connected via the Yangtze (*N* = 8) and Yellow (*N* = 2) river systems and two populations from isolated plateau lakes (*N* = 2). For all populations, we genotyped two genetic marker systems with different evolutionary speeds: the mitochondrial *COI* (cytochrome oxidase I) gene and seven microsatellite loci. We used the data to estimate genetic diversity and differentiation and effective population sizes and to explore the demographic history of local populations. Specifically, we aimed to differentiate between two main competing scenarios and their potential underlying evolutionary mechanisms: (1) limited genetic divergence and high intrapopulation diversity of local populations would suggest large population sizes and good historic connectivity, (2) In contrast, low genetic diversity and strong differentiation of local populations could be the result of a sessile life style, the occurrence in lacustrine systems, large geographic distances isolating populations, and the proposed strong anthropogenic pressure (overharvesting and fragmentation).

## Materials and Methods

### Sampling

**Figure 1:**
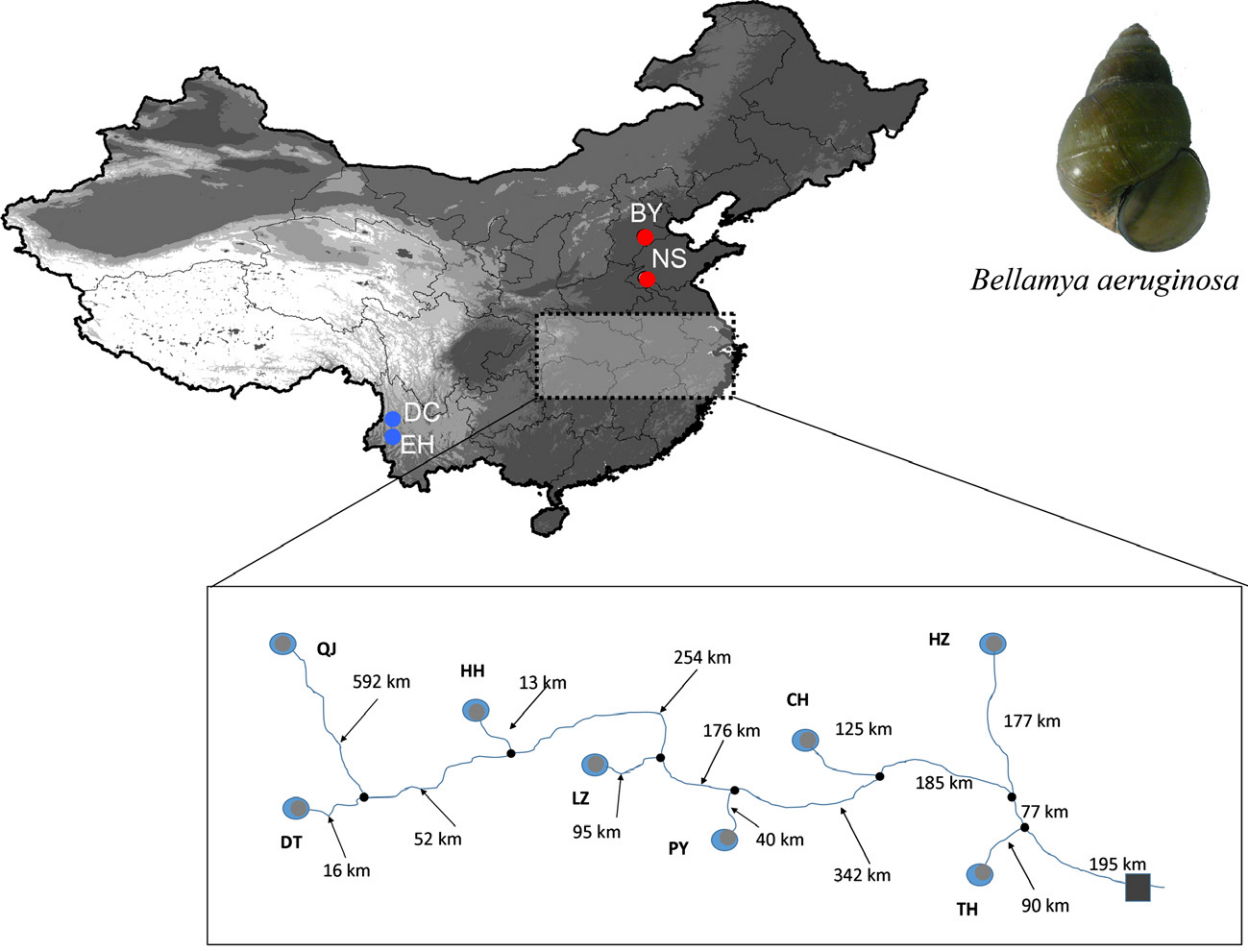
Sampling map with sampling location codes (Table 1) displaying the study region in China; red – sampling locations in the Yellow River system, gray – sampling locations at the Yangtze River system, and blue – sampling locations at plateau lakes. The box shows the position of sampling locations within the Yangtze River, numbers along the stream represent distances.

*Bellamya aeruginosa* populations were sampled from 10 interconnected lakes in the Yellow (*N* = 2) and Yangtze (*N* = 8) river systems and two isolated Plateau lakes (*N* = 2) in China (Fig. 1 and Table 1). A total of 277 individuals were obtained between October 2011 and July 2012. Species identification followed the keys provided by Zhang and Liu (1960). Adductor muscles were preserved in 95 % ethanol and stored at −20°C until DNA extraction.

**Table 1:** Sampling information and general statistics for both genetic markers: N (usats), number of individuals for microsatellite analysis; *N*_A_, number of alleles; *H*_O_, observed heterozygosity; *H*_E_, expected heterozygosity; *F*_IS_, inbreeding coefficient; N (COI) number of individuals for mitochondrial analysis; S, number of polymorphic sites; nh, number of haplotypes; h, haplotype diversity; *π*, nucleotide diversity

ID	Location	River	Coordinates	N (usats)	*N*_A_	*H*_O_	*H*_E_	*F*_IS_	N (*COI*)	S	*nh*	h	*π*
BY	Baiyangdian Lake	Yellow	N38°52′57.67″ E116°03′44.77″	21	11.7	0.659	0.785	0.184	9	63	9	0.978	0.033
NS	Nansi Lake	Yellow	N34°40′10.88″ E117°16′13.05″	24	11.7	0.694	0.820	0.175	10	70	8	0.933	0.034
CH	Chaohu Lake	Yangtze	N31°39′28.75″ E117°17′41.99″	30	14.7	0.760	0.860	0.132	10	77	9	0.978	0.037
DT	Dongting Lake	Yangtze	N29°21′16.60″ E113°00′30.72″	24	14.3	0.740	0.857	0.159	9	21	7	0.944	0.013
HH	Honghu Lake	Yangtze	N29°54′28.05 ″E113°27′50.75″	20	14.3	0.782	0.857	0.113	10	68	9	0.978	0.040
HZ	Hongze Lake	Yangtze	N33°18′20.30″ E118°50′35.03″	20	12.4	0.719	0.830	0.159	9	61	8	0.972	0.028
LZ	Liangzi Lake	Yangtze	N30°21′06.18″ E114°26′17.13″	25	12.7	0.757	0.853	0.133	10	45	7	0.867	0.022
PY	Poyang Lake	Yangtze	N29°26′26.30″ E116°02′25.5″	24	13.9	0.717	0.837	0.165	9	38	6	0.889	0.015
QJ	Qingjing River	Yangtze	N30°15′48.44″ E109°28′41.16″	24	11.7	0.657	0.833	0.233	11	77	9	0.964	0.045
TH	Taihu Lake	Yangtze	N31°26′58.67″ E120°00′57.87″	23	14.1	0.749	0.864	0.156	10	75	7	0.867	0.035
DC	Dianchi Lake	Plateau Lake	N24°57′44.80″ E102°39′05.20″	20	11	0.709	0.832	0.173	10	75	6	0.889	0.034
EH	Erhai Lale	Plateau Lake	N25°36′26.35″ E100°14′26.84″	22	11.3	0.734	0.808	0.116	9	41	6	0.889	0.016

### Mitochondrial *COI* sequencing

Genomic DNA was extracted with the DNeasy Blood and Tissue Kit (QIAGEN, Hilden, Germany) following the manufacturer's protocol and conserved at −20°C until further use. We amplified partial *COI* sequences using the universal primers for invertebrates described by Folmer et al. (1994). PCR products were purified following a standard sodium acetate precipitation (Bürgmann et al. 2001). A total of 116 specimens were sequenced using the BigDye Terminator kit v. 3.1 (Applied Biosystems, Foster City, CA) and were processed by Sangon Biotech Co., Ltd. (Shanghai, China). All individuals were sequenced in both directions. Forward and reverse sequences were inspected and aligned using the SEQMAN software in Lasergene v. 7.1 (DNAstar, Madison, WI). Consensus sequences were blasted to confirm that the sequence amplified was the correct target DNA fragment. All new sequences were deposited in GenBank (accession numbers: KF535413‐KF535429, KF535431‐KF535468, KP150575‐KP150635).

### *COI* analysis

The resulting 116 sequences were aligned with Clustal X (Thompson et al. 1997). Variable sites, parsimony informative sites, number of haplotypes, and haplotype (*h*) and nucleotide diversity (*π*) were calculated with DNASP v. 5 (Librado and Rozas 2009). ARLEQUIN v. 3.5 (Excoffier and Lischer 2010) was used to test for neutrality employing Tajima's *D* (Tajima 1989) and Fu's *F* statistics (Fu 1997). AMOVA (Analysis of molecular variance) was conducted in ARLEQUIN to partition the genetic variance within and among populations. Furthermore, three groups were defined according to river system (NS and BY as Yellow River system, EH and DC as Plateau Lake; all other populations as Yangtze River system) to estimate variance components. Pairwise Φ_ST_ values were calculated to assess population differentiation. We generated a statistical parsimony haplotype network with a 95 % connection limit with TCS v. 1.2.1 (Clement et al. 2000). Further, we used the *COI* data to generate Bayesian skyline plots to explore the demographic history of each population alone and the whole combined data set. This method permits the reconstruction of past population demography and generates plots of female effective population sizes (*N*_e_) over time. The appropriate substitution model was determined as HKY+I+G with jModeltest v. 2 (Darriba et al. 2012). We created individual input files for each population, and all populations combined with BEAUti v.1.7.4 (implemented in the BEAST package). Analyses were run in BEAST v.1.7.4 (Drummond et al. 2012). We used a strict clock with a published substitution rate of 1.32 % per million years for invertebrates estimated for *COI* under the HKY model (Wilke et al. 2009). The program was run for 10 million generations sampling every 10,000 generations. The Bayesian skyline plots were subsequently generated with Tracer v. 1.5 (Rambaut and Drummond 2009). We tested for isolation by linear geographic distance across all data. As sampling at different geographic scales may lead to artificial IBD patterns, we also tested for isolation by distance and stream distance within the Yangtze River only using a Mantel test with 10,000 randomizations as implemented in IBD v. 1.52 (Bohonak 2002). Stream distances were measured along the streams between sites using ArcMap v. 10 (ESRI 2011).

### Microsatellite markers analysis

All samples (*N* = 277) were genotyped at seven microsatellite loci (Table S1). The PCR amplifications were performed in a total volume of 10 *μ*L: 1× PCR buffer (100 mmol/L Tris–HCl, 500 mmol/L KCl), 20–40 ng genomic DNA, 0.5 mmol/L of each primer, 200 mmol/L of each dNTP, 1.5 mmol/L MgCl_2_, and 0.25 U of Taq DNA polymerase (TaKaRa, Dalian, China). Thermal cycling was performed with a TProfessional Thermocycler (Biometra, Göttingen, Germany) under the following conditions: 94°C for 5 min, 35 cycles at 94°C for 45 sec, annealing at 50–55°C depending on the marker for 30 sec (details in Table S1), 72°C for 30 sec, and a final extension at 72°C for 10 min. Forward primers were 5′‐labeled with HEX, ROX, or TAMRA (Table S1). The sizes of the fluorescently labeled PCR products were estimated according to an internal size marker (LIZ‐500) on an ABI PRISM 3700 sequencer (Applied Biosystems). Fragment lengths were scored using STRAND v. 2.3.48 (UC Davis Veterinary Genetics Laboratory, Davis, CA).

The resulting data were first inspected with MICRO‐CHECKER v. 2.2.3 (Van Oosterhout et al. 2004) to test for unexpected mutation steps, large gaps, unusually sized alleles and the presence of null alleles. The number of alleles (*N*_A_), the expected (*H*_*E*_) and observed heterozygosities (*H*_*O*_), *F*_ST_, and the inbreeding coefficients *F*_IS_ (Weir and Cockerham 1984) were estimated with MSA (MICROSATELLITE ANALYSER) v. 3.15 (Dieringer and Schlötterer 2003). GENEPOP v. 4.0.10 (Rousset 2008) was used to measure heterozygote deficiency or excess and to assess deviations from HWE (Hardy–Weinberg equilibrium). *P‐*values were corrected for multiple comparisons by applying a sequential Bonferroni correction (Rice 1989). We tested for recent bottlenecks events using BOTTLENECK v. 1.2.02 (Piry et al. 1999) under the TPM (two‐phased model), with the proportion of stepwise mutations set to 95 % and the variance set at 15. Significance of deviations was tested using the Wilcoxon sign‐rank test with 1000 iterations. Further, we used the microsatellite data to calculate effective population sizes (*N*_e_). We used LD*N*_e_ as implemented in *N*eEstimator v. 2 (Do et al. 2014) to calculate *N*_e_ considering results from 0.02 and 0.01 as lowest allowed allele frequencies.

Pairwise migration rates between the 12 populations were estimated using a maximum likelihood coalescent approach implemented in MIGRATE v 3.0 (Beerli and Felsenstein 2001); we estimated Ө and M (immigration rate/mutation rate) based on *F*_ST_ values. We ran 10 short chains with a total of 10,000 genealogy samples and three long chains with 1,000,000 samples, following a burn‐in of 10,000 samples; three independent runs were performed. As MIGRATE has been suggested to be vulnerable to violations of the assumption of stable population sizes, we also used the Bayesian approach developed by Wilson and Rannala implemented in BAYESSASS 3.0 (Wilson and Rannala 2003) to infer migration rates. The program uses genotypic data and MCMCs (Markov Chain Monte Carlo) to infer recent patterns of gene flow. We performed five independent analyses. Each run contained 35,000,000 MCMC iterations, with a burn‐in of 3,500,000 iterations and a sampling frequency of 3500 generations with a random seed. To ensure sufficient mixing of the MCMCs and to improve the coverage of the probability space, we adjusted the acceptance rate for estimated allele frequencies and inbreeding coefficients. We increased the mixing parameters for the allele frequencies (ΔA) to 0.50 and for the inbreeding coefficient (ΔF) to 0.80. Mixing and convergence of MCMCs were visually assessed using TRACER. From the five independent runs, we chose the run with the lowest Bayesian deviance in the logProb calculated using the R‐function (R Development Core Team 2009) provided by Faubet et al. (2007) and as suggested in Meirmans (2014).

An analysis of molecular variance was performed with ARLEQUIN to partition the genetic variance among and within populations, similar to the mtDNA data. We also tested for isolation by linear geographic distance and stream distance (see mtDNA data) using Mantel tests with 10,000 randomizations as implemented in IBD v. 1.52 (Bohonak 2002). Population genetic structure was further analyzed using the Bayesian algorithm implemented in STRUCTURE v. 2.3 (Pritchard et al. 2000). The software was used to cluster individuals into K populations. The number of genetic clusters (*K*) was assessed assuming an admixture ancestry model with correlated allele frequencies; 20 independent runs were performed with 500,000 MCMC repetitions at each run discarding a burn‐in of 150,000 iterations. We tested K from 1 to 13 and used the ad‐hoc statistic Δ*K* (Evanno et al. 2005) to determine the most likely value of *K*. Data were sorted with CLUMPP v. 1.1.2 (Jakobsson and Rosenberg 2007), and aligned data were visualized in DISTRUCT (Rosenberg 2004).

## Results

### Genetic variation – *COI*

We generated 116 *COI* sequences with a length of 709 bp, 81 of which represented unique haplotypes. The alignment contained 167 variable sites (23.6 %), 136 of which were parsimony informative. Nucleotide (*π*) and haplotype diversities (*h*) across all populations were 0.038 and 0.985, respectively. Tajima's *D* and Fu's *Fs* were not significant in any population.

### Genetic variation – microsatellite markers

No evidence of scoring errors was revealed by MICRO‐CHECKER. However, the presence of null alleles was suggested for several loci (Table S2). However, no systematic distribution of null alleles across populations was found and only few populations were affected; therefore, all loci were maintained for further analyses. GENEPOP showed deviations from HWE for all populations at multiple loci even after Bonferroni correction (*P *<* *0.007). Probability values for *H*_E_ excess were > 0.813 for all populations except QJ (Qingjiang River) (*P *=* *0.679) (Table 1). The number of alleles (*N*_A_) ranged from 5 to 25 across the seven microsatellite loci. The mean *N*_A_ across all loci ranged from 11.0 for Dianchi Lake (DC) to 14.7 for Chaohu Lake (CH) (Table 1). Observed heterozygosity (*H*_O_) and expected heterozygosity (*H*_E_) were overall high across all populations with mean values ranging from 0.657 to 0.782 and 0.785 to 0.864, respectively (Table 1). Inbreeding coefficients ranged from 0.113 to 0.233 (Table 1). Most populations showed no signs of bottlenecks, except for Honghu Lake (HH) (*P *=* *0.008) and QJ (*P *=* *0.016), suggesting that most studied populations were under mutation–drift equilibrium.

### Population structure

*F*_ST_ estimates from the microsatellite data ranged from 0.018 to 0.107; Φ_ST_ estimates from the mitochondrial sequences ranged from 0.002 to 0.133 (data not shown). Most Φ_ST_ and all *F*_ST_ values were low and not statistically significant (*P *>* *0.05). AMOVA of the microsatellite data revealed that 93.31 % of genetic variation was explained by intrapopulation variation, while the remaining 6.69 % (*P *<* *0.05) explained variation among populations. For mitochondrial sequences, the amount of variance explained by differences between populations was similar (6.03 %) (Table 2). Another AMOVA was conducted using river systems as groups (NS and BY as Yellow River system, EH and DC as Plateau Lakes; all other populations as Yangtze River system). However, very low differentiation at the group level was found for both microsatellite and *COI* data (Table 3).

**Table 2:** Analysis of molecular variance partitioning the genetic variance within and among *Bellamya aeruginosa* populations for microsatellite and mtDNA data

Source of variation	df	Sum of squares	Variance components	Percentage of variation with *P* value
Microsatellites				
Among populations	11	135.680	0.205	6.69 (*P *=* *0.000)
Within populations	542	1551.840	2.863	93.31 (*P *=* *0.000)
Total	553	1687.520	3.069	
mtDNA				
Among populations	11	434.133	2.989	6.03 (*P *=* *0.000)
Within populations	104	1100.315	10.580	93.97 (*P *=* *0.000)
Total	115	1534.448	13.569	

**Table 3:** Analysis of molecular variance results comparing genetic variation in *Bellamya aeruginosa* collected from 12 lakes among three geographic regions (NS and BY grouped as Yellow River, EH and DC grouped as Plateau lakes, all other populations grouped as Yangtze River)

Source of variation	df	Sum of squares	Variance components	Percentage of variation with *P* value
Microsatellites				
Among groups	2	50.470	0.113	3.60 (*P *=* *0.001)
Among populations within groups	9	94.682	0.152	5.22 (*P *=* *0.000)
Within populations	265	910.602	0.552	91.18 (*P *=* *0.000)
				
mtDNA				
Among groups	2	72.967	−0.895	−0.23 (*P *=* *0.544)
Among populations within groups	9	517.189	4.491	6.16 (*P *=* *0.000)
Within populations	104	1452.226	13.964	94.07 (*P *=* *0.000)
				

**Figure 2:**
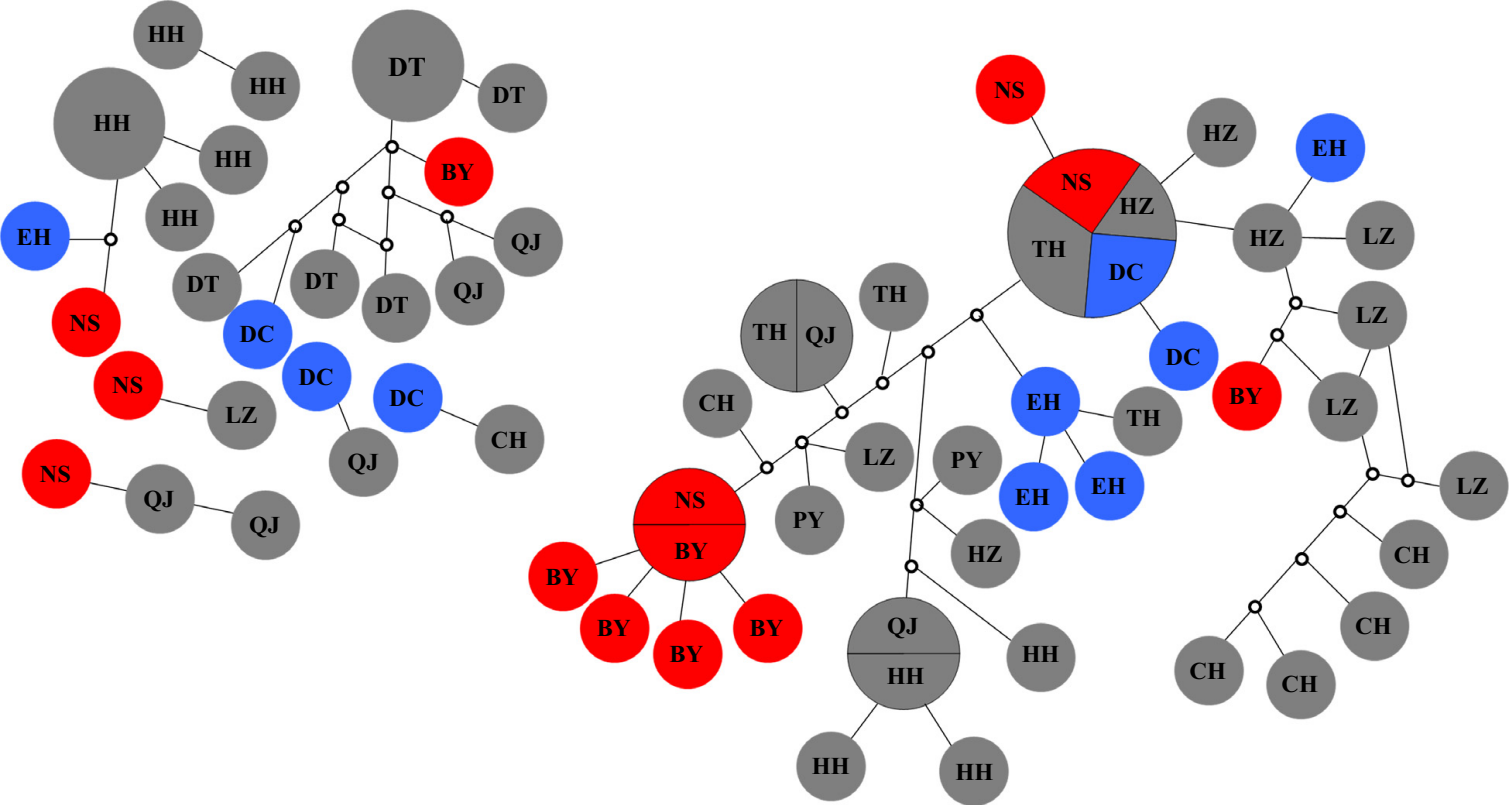
Statistical parsimony haplotype network with a 95 % connection limit for 116 COI sequences (709 bp) of *Bellamya aeruginosa* constructed with TCS. The size of each circle indicates the frequency of the respective haplotype; unsampled haplotypes are indicated by small empty circles. Red represents haplotypes from the Yellow River system, gray represents haplotypes from the Yangtze River system and blue represents haplotypes from plateau lakes.

**Figure 3:**
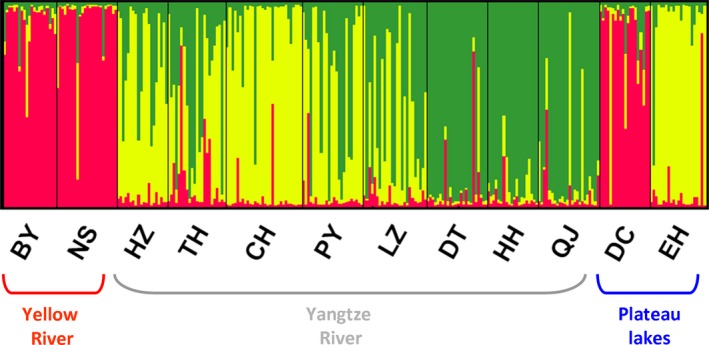
Structure plot for the 12 *Bellamya aeruginosa* populations based on the microsatellite data (shown for the most likely *K* = 3). The sampling location codes (Table 1) are indicated along the *x*‐axis. Each vertical line represents one individual, and y‐coordinates denote each individual's percentage assignment to each of the three genetic clusters.

Mitochondrial haplotypes from the same sampling locations did not cluster together with the exception of CH (Chaohu Lake, Yangtze River system), BY (Baiyangdian Lake, Yellow River system) and HH populations (Honghu Lake, Yangtze River system) (Fig. 2). STRUCTURE analysis of the mitochondrial data indicated a grouping into three genetic clusters (Δ*K* = 67.3 for *K *=* *3, Figs 3). Most of the individuals from NS, BY, and DC were assigned to the first genetic cluster, EH and CH represented the second cluster, and the populations from DT, HH, and QJ represented a third cluster, while other populations tended to be more admixed (HZ, TH, PY, LZ).

**Figure 4:**
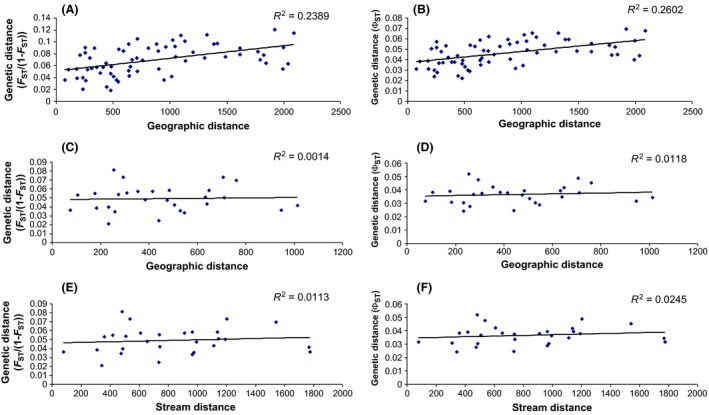
Isolation by linear distance across all populations for (A) microsatellite data, and (B) mitochondrial data, and only for the Yangtze River for (C) microsatellite data, and (D) mitochondrial data; isolation by stream distance for the Yangtze River using (E) microsatellite data and (F) mitochondrial data.

We found a significant isolation by linear distance pattern for the microsatellite data (Mantel test, *r* = 0.4888; *P *=* *0.011, Fig. 4A) and *COI* data (*r* = 0.5101; *P *=* *0.011, Fig. 4B), when all populations were included. When we only analyzed the populations from the Yangtze catchment, no isolation by linear or stream distance was detected for either data set (Fig. 4C–F).

### Gene flow

Gene flow estimates based on the microsatellite data calculated with MIGRATE and BAYESASS indicated moderate to high levels of gene flow between populations (Tables 4 and 5). MIGRATE estimated the effective number of migrants entering and leaving each population per generation (*M*) between 1.29 (QJ→DT) and 74.76 (LZ→QJ) (Table 4). Most of populations had asymmetrical gene flow except for HZ, NS, and DC populations. The highest unidirectional estimates of gene flow estimated by MIGRATE were found toward the HH population (*M *=* *188.47) and the lowest was for CH (*M *=* *90.03), while the highest gene flow out of a population was from LZ (*M *=* *251.94). Similarly, many migration rate estimates from BAYESASS were moderate to high, ranging from 0.01 to 0.237 (76 of 144 values); the remainder varied from 0.008 to 0.009 (Table 5).

**Table 4:** Immigration rates (M) into each of the 12 *Bellamya aeruginosa* populations from every other population as estimated by MIGRATE

pop		CH	DT	PY	QJ	HZ	NS	BY	TH	LZ	HH	EH	DC	Total→i
*i*	Θ_*i*_	CH→*i*	DT→i	PY→i	QJ→i	HZ→i	NS→i	BY→i	TH→i	LZ→i	HH→i	EH→i	DC→i	
CH	0.0941		7.16	3.69	9.56	6.41	6.33	9.68	19.38	3.49	4.29	15.55	4.50	90.03
DT	0.0953	13.95		10.70	1.29	16.00	3.57	4.75	17.56	18.04	17.68	10.20	8.17	107.97
PY	0.0662	11.88	5.44		5.71	7.41	3.78	11.16	6.81	29.65	9.46	10.23	4.93	94.57
QJ	0.0468	11.37	10.59	9.45		8.43	19.77	5.12	6.84	74.76	17.13	13.08	12.45	177.61
HZ	0.0827	35.47	7.44	18.47	1.48		10.59	25.02	13.05	8.93	13.43	7.72	8.33	114.46
NS	0.0920	9.59	4.99	15.03	2.41	5.55		30.59	4.46	27.47	8.43	19.06	13.58	131.56
BY	0.0949	11.79	6.29	13.58	12.07	7.29	13.45		5.90	16.45	5.35	13.48	11.18	105.04
TH	0.0958	12.83	12.05	35.68	19.12	16.51	13.23	7.30		4.22	8.06	7.90	2.07	126.12
LZ	0.0608	8.71	3.51	14.32	6.68	13.73	9.41	4.48	11.84		9.03	17.22	12.57	102.78
HH	0.0947	10.38	17.19	21.74	7.26	11.96	10.62	20.21	15.06	54.26		11.80	18.38	188.47
EH	0.0855	39.47	8.07	28.56	10.82	11.84	19.84	20.18	51.26	9.98	12.84		6.18	179.55
DC	0.0261	7.20	10.76	16.69	5.16	5.90	11.78	8.17	15.62	4.70	12.78	6.83		98.39
Total		172.63	93.49	187.92	81.54	111.03	122.35	146.65	167.78	251.94	118.48	133.06	102.34	

**Table 5:** Estimates of migration rates (proportion of individuals) among populations of *B. aeruginosa*, derived by BAYESASS

		Migration from											
		BY	NS	HZ	TH	CH	PY	LZ	DT	HH	QJ	DC	EH
Migration into	BY	0.678 (0.657–0.698)	0.225 (0.173–0.277)	0.009 (0.000–0.026)	0.009 (0.000–0.027)	0.009 (0.000–0.026)	0.009 (0.000–0.026)	0.017 (0.000–0.041)	0.009 (0.000–0.026)	0.009 (0.000–0.026)	0.009 (0.000–0.026)	0.009 (0.000–0.026)	0.009 (0.000–0.026)
	NS	0.009 (0.000–0.027)	0.890 (0.837–0.944)	0.009 (0.000–0.027)	0.011 (0.000–0.032)	0.010 (0.000–0.030)	0.009 (0.000–0.0.27)	0.012 (0.000–0.032)	0.009 (0.000–0.027)	0.009 (0.000–0.027)	0.009 (0.000–0.027)	0.010 (0.000–0.030)	0.010 (0.000–0.030)
	HZ	0.009 (0.000–0.027)	0.009 (0.000–0.027)	0.678 (0.657–0.699)	0.212 (0.153–0.271)	0.019 (0.000–0.050)	0.009 (0.000–0.026)	0.010 (0.000–0.029)	0.010 (0.000–0.028)	0.009 (0.000–0.026)	0.009 (0.000–0.027)	0.010 (0.000–0.029)	0.015 (0.000–0.039)
	TH	0.010 (0.000–0.029)	0.021 (0.000–0.058)	0.010 (0.000–0.028)	0.846 (0.0779–0.913)	0.019 (0.000–0.052)	0.010 (0.000–0.028)	0.015 (0.000–0.044)	0.012 (0.000–0.035)	0.010 (0.000–0.028)	0.010 (0.000–0.029)	0.020 (0.000–0.053)	0.018 (0.000–0.050)
	CH	0.008 (0.000–0.024)	0.008 (0.000–0.024)	0.008 (0.000–0.023)	0.044 (0.000–0.093)	0.857 (0.0797–0.916)	0.008 (0.000–0.024)	0.012 (0.000–0.033)	0.012 (0.000–0.034)	0.008 (0.000–0.023)	0.008 (0.000–0.023)	0.015 (0.000–0.038)	0.012 (0.000–0.034)
	PY	0.008 (0.000–0.023)	0.009 (0.000–0.025)	0.008 (0.000–0.023)	0.237 (0.189–0.285)	0.009 (0.000–0.026)	0.676 (0.658–0.694)	0.010 (0.000–0.030)	0.008 (0.000–0.024)	0.008 (0.000–0.024)	0.008 (0.000–0.024)	0.009 (0.000–0.026)	0.009 (0.000–0.025)
	LZ	0.009 (0.000–0.026)	0.009 (0.000–0.027)	0.009 (0.000–0.026)	0.051 (0.000–0.115)	0.016 (0.000–0.045)	0.009 (0.000–0.026)	0.833 (0.754–0.912)	0.018 (0.000–0.047)	0.009 (0.000–0.027)	0.009 (0.000–0.026)	0.009 (0.000–0.027)	0.018 (0.000–0.047)
	DT	0.009 (0.000–0.027)	0.012 (0.0000–0.033)	0.010 (0.000–0.028)	0.034 (0.000–0.00.89)	0.015 (0.000–0.040)	0.010 (0.000–0.028)	0.021 (0.000–0.052)	0.851 (0.783–0.920)	0.009 (0.000–0.027)	0.010 (0.000–0.028)	0.011 (0.000–0.031)	0.010 (0.000–0.028)
	HH	0.009 (0.000–0.026)	0.010 (0.000–0.027)	0.009 (0.000–0.027)	0.046 (0.000–0.099)	0.010 (0.000–0.029)	0.010 (0.000–0.028)	0.009 (0.000–0.028)	0.191 (0.125–0.258)	0.678 (0.657–0.689)	0.009 (0.000–0.026)	0.010 (0.000–0.270)	0.010 (0.000–0.028)
	QJ	0.008 (0.000–0.024)	0.009 (0.000–0.025)	0.008 (0.000–0.025)	0.198 (0.130–0.027)	0.008 (0.000–0.024)	0.008 (0.000–0.024)	0.046 (0.000–0.104)	0.012 (0.000–0.033)	0.008 (0.000–0.024)	0.677 (0.658–0.695)	0.008 (0.000–0.024)	0.009 (0.000–0.025)
	DC	0.011 (0.000–0.031)	0.015 (0.000–0.042)	0.011 (0.000–0.031)	0.014 (0.000–0.039)	0.018 (0.000–0.047)	0.011 (0.000–0.030)	0.014 (0.000–0.0039)	0.011 (0.000–0.031)	0.011 (0.000–0.031)	0.011 (0.000–0.031)	0.865 (0.805–0.925)	0.011 (0.000–0.033)
	EH	0.010 (0.000–0.028)	0.013 (0.000–0.036)	0.010 (0.000–0.028)	0.027 (0.000–0.072)	0.012 (0.000–0.034)	0.010 (0.000–0.028)	0.012 (0.000–0.033)	0.010 (0.000–0.030)	0.010 (0.000–0.029)	0.010 (0.000–0.029)	0.015 (0.000–0.043)	0.863 (0.795–0.863)

Means of the posterior distributions of m, the migration rate into each population, are shown. The populations from which each individual was sampled are listed in the rows, while their populations from which they migrated are listed in the columns. Values along the diagonal are the proportions of individuals derived from the source populations each generation. Values in parentheses below migration rates are 95% CI.

### Current and historical demography

The effective population size estimates derived from LD*N*_e_ analyses were not very precise, appeared to vary between populations, yet were mostly large (estimated as infinite for five of the 12 populations, range of other populations from 55 for CH to 1006 for YX).

BOTTLENECK analyses of the microsatellite data revealed no evidence of a recent bottleneck for most of populations except for the HH and QJ populations. No significant heterozygosity excess was detected in any locality under the TPM.

**Figure 5:**
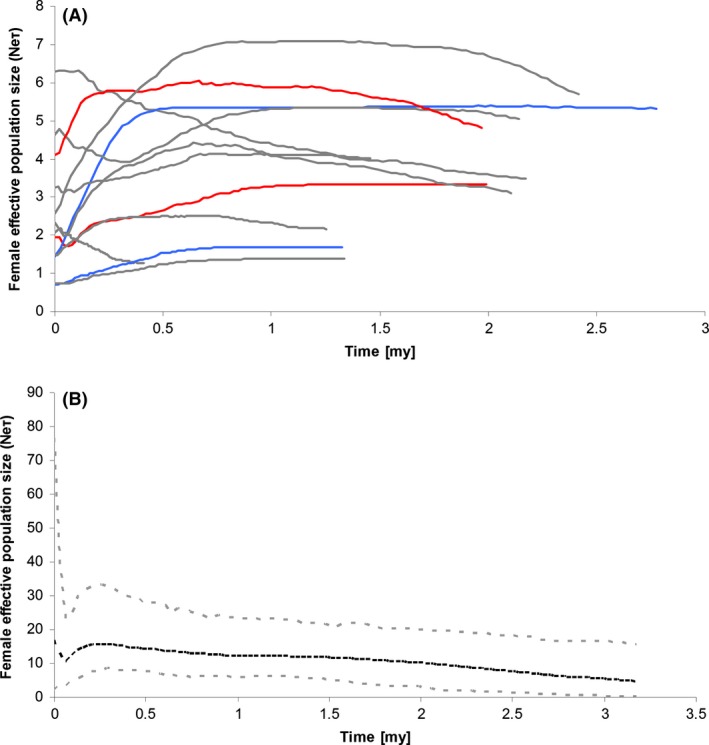
BSP (Bayesian Skyline Plots) generated using the cytochrome oxidase I sequences and applying a fixed mutation rate of 1.34% sequence divergence per million years and the HKY + I + G model as determined by jModeltest. BSPs were generated for (A) each population and (B) for the species as a whole. Red – Yellow River, gray – Yangtze River, blue – plateau lakes. Dotted line – all populations combined , grey dotted line – 95 % HPD.

The Bayesian skyline plots detected relatively stable population sizes throughout the last 2 mya for most populations (Fig. 5). The overall population size of the species in the study region appears to have increased constantly from 3 mya to 300 kya, when the population sizes strongly dropped. Subsequent to this drop, population size recovered quickly even extending predecline population size.

## Discussion

Our population genetic analysis of *B. aeruginosa* supports our first scenario: we found high levels of genetic diversity and strong population admixture across the 12 studied populations of *B. aeruginosa* for both mitochondrial and nuclear markers despite a sessile life style and large separating geographic distances. Current population sizes appear to be mostly large, which was also supported by the high genetic diversity and past fluctuations of population sizes appear to be limited. However, the species as a whole seems to have rapidly declined during the mid‐ to Late Pleistocene and postglacially expanded. Yet, no recent bottlenecks were detected with the microsatellite data. Our findings suggest that gene flow is moderate to high among the sampling locations, even between river systems despite vast geographic distances and low active dispersal ability. The results further indicate that the species at the moment can maintain high genetic diversity despite strong harvesting pressure and increasing fragmentation. These findings may be explained by a combination of large population sizes with high standing genetic variation and long‐distance gene flow, potentially facilitated by zoochoric and anthropogenic dispersal. In the following, we discuss each of these findings in detail.

### Genetic diversity and current and past population demography

Both marker systems revealed high genetic diversity and low levels of genetic differentiation between local populations (Tables 1, 2, 3, 4, Figs. 2, 3). Current population sizes were generally large and confidence intervals in all, but one population included infinity. Population sizes of single populations appeared to be relatively stable with increases or declines occurring within the last 500 ka, independent of the stream system (Fig. 5). Overall, the population size of all combined populations constantly increased starting 3 mya with a sudden drop around 300 kya. Within the last 100 ka, the population sizes increased again even exceeding the predecline size (Fig. 5).

Our findings suggest that the demographic history of the species has been influenced by past climatic cycles with a drop in population size corresponding to the Late Pleistocene and postglacial range expansion (Yang et al. 2009; Bagley et al. 2013). For ten populations, we detected a drop in population size from mid‐ or Late Pleistocene (Fig. 5). The Tali glacial stage of the Late Pleistocene (10–110 ka) likely had a great influence on the distribution of *Bellamya* in plateau lakes, while the Taku glaciation in the early Mid‐Pleistocene and the Lushan glaciation in the Late Mid‐Pleistocene (200–230 ka) (Wissmann 1937; Tang and Tao 1997; Xiang et al. 2006) may have led to a fast decline of *Bellamya* populations across the Yangtze River system. At more recent time scales, microsatellites could not detect bottlenecks for most populations suggesting no recent drastic population declines. The current population sizes are likely large and together with high migration rates allow for the maintenance of high genetic diversity. These findings of high genetic diversity and little differentiation is comparatively uncommon in freshwater snails in lakes due to their low migration ability, high self‐fertilization rates, and historical demographic instability (Campbell et al. 2010; Nalugwa et al. 2011; Standley et al. 2014). However, *Bellamya* is somewhat special as the genus has a relatively short generation time of only 4 months (Ma et al. 2010), which in combination with their specific mating strategy (gonochorism and viviparity), may make them less vulnerable to rapid loss of genetic diversity resulting from population fluctuations and geographic isolation (Li et al. 2011; Tian‐Bi et al. 2013). Further, the lacustrine habitats may represent relatively stable habitats, which allow for large stable population sizes, further helping to maintain high genetic diversity. Hence, the combination of the environmental setting and species specific life‐history traits together promotes the maintenance of large stable populations in *B. aeruginosa*.

### Low population divergence and weak geographic structure

Another aspect potentially contributing to the high genetic diversity are high migration rates, even between far distant populations. We found that the study populations are only slightly differentiated from each other. *F*_ST_ and Φ_ST_ values were low and mostly insignificant. We found a significant signal of isolation by distance for both genetic markers across the whole study region (Fig. 4A and B). Isolation by distance and isolation by stream distance in the Yangtze River, however, were much weaker (Fig. 4C–F). AMOVA showed limited divergence between populations and regions for both data sets (Tables 2). Bayesian inference of recent migration rates suggested consistent, intermediate to high levels level of gene flow between populations (Table 5). The haplotype network for the mitochondrial data showed little sorting of locations (Fig. 2) and STRUCTURE suggested three lineages; yet, these only partially correspond to the stream systems and geographic regions.

All these findings together suggest that gene flow is moderate to high and not only occurs within streams, but also between different drainages (Yangtze River and Yellow River); even isolated plateau lakes receive migrants. Within the Yangtze River for which we have the largest number of samples, no strong isolation by stream distance was detected, suggesting that populations along the stream frequently exchange individuals. The absence of significant isolation by distance and weak geographic signal among populations is not uncommon in snail species (Wada et al. 2012). This may at least in riverine systems partially be due to frequent flooding events during which specimens may be swept away and transported even to far distant locations within the stream (Van Leeuwen et al. 2013). Accidental transport during flooding may change population genetic patterns within rivers and even completely remove isolation by distance patterns (Crispo and Chapman 2009). Such flash floods are common in the Yangtze River catchment, and all of the lakes sampled in this catchment are connected to the river.

Flooding, however, cannot explain the strong admixture between far distant sampling locations across different river systems and isolated plateau lakes (as seen e.g., in Figs. 2, 3). Here, anthropogenic translocations and animal‐mediated dispersal via waterfowl represent a possible explanation, a commonly observed phenomenon in freshwater snails (Green and Figuerola 2005; Gittenberger et al. 2006; Gittenberger 2012; Kopp et al. 2012). Human‐ and animal‐mediated dispersal, for example, explains the present day distribution of *Isabellaria buresi pharsalica* in Thessaly (Uit de Weerd et al. 2005). As *Bellamya* are commonly harvested by humans, anthropogenic translocations appear likely for this genus (Prabhakar and Roy 2009; Li et al. 2010). Such translocations, independent of the vector, appear to have positive effects on the genetic diversity of the species.

## Conclusion

Our genetic analysis revealed high levels of intrapopulation genetic variation, large effective population sizes, moderate to high levels of gene flow, and low interpopulation differentiation. The high rates of gene flow within the Yangtze catchment may be supported by drifting during frequent flooding events, while gene flow between different catchments likely is facilitated by animal‐ or human‐mediated dispersal. All together, the conservation status of the species seems currently uncritical despite strong harvesting pressure and anthropogenic habitat fragmentation.

## Conflict of Interest

None declared.

## Supporting information


**Figure S1.** Distributions of pairwise nucleotide differences (mismatch distributions) of COI gene in *B. aeruginosa* samples and the corresponding Fu's *Fs* value.
**Figure S2.** The most likely value of *K* (inferred cluster) estimated using Evanno's ΔK‐method.

**Table S1.** Primer sequence for microsatellite markers and optional PCR conditions.
**Table S2.** The frequencies of null alleles across seven microsatellite loci and 12 geographic locations for *B. aeruginosa*, as suggested by the program MICRO‐CHECKER.

## References

[ece31673-bib-0002] Bagley, J. C. , M. Sandel , J. Travis , M. de Lourdes Lozano‐Vilano , and J. B. Johnson . 2013 Paleoclimatic modeling and phylogeography of least killifish, *Heterandria formosa*: insights into Pleistocene expansion‐contraction dynamics and evolutionary history of North American Coastal Plain freshwater biota. BMC Evol. Biol. 13:223. doi:10.1186/1471‐2148‐13‐223. 24107245 10.1186/1471-2148-13-223 PMC3851817

[ece31673-bib-0003] Beerli, P. , and J. Felsenstein . 2001 Maximum likelihood estimation of a migration matrix and effective population sizes in n subpopulations by using a coalescent approach. Proc. Natl Acad. Sci. USA 98:4563–4568. 11287657 10.1073/pnas.081068098 PMC31874

[ece31673-bib-0005] Bohonak, A. J. 2002 IBD (isolation by distance): a program for analyses of isolation by distance. J. Hered. 93:153–154. 12140277 10.1093/jhered/93.2.153

[ece31673-bib-0006] Bürgmann, H. , M. Pesaro , F. Widmer , and J. Zeyer . 2001 A strategy for optimizing quality and quantity of DNA extracted from soil. J. Microbiol. Methods 45:7–20. 11295193 10.1016/s0167-7012(01)00213-5

[ece31673-bib-0007] Campbell, G. , L. R. Noble , D. Rollinson , V. R. Southgate , J. P. Webster , and C. S. Jones . 2010 Low genetic diversity in a snail intermediate host (*Biomphalaria pfeifferi* Krass, 1848) and schistosomiasis transmission in the Senegal River Basin. Mol. Ecol. 19:241–256. 20025653 10.1111/j.1365-294X.2009.04463.x

[ece31673-bib-0008] Chaine, N. M. , C. R. Allen , K. A. Fricke , D. M. Haak , M. L. Hellman , R. A. Kill , et al. 2012 Population estimate of Chinese mystery snail (*Bellamya chinensis*) in a Nebraska reservoir. Bioinv. Rec. 1:283–287.

[ece31673-bib-0009] Clement, M. , D. Posada , and K. A. Crandall . 2000 TCS: a computer program to estimate gene genealogies. Mol. Ecol. 9:1657–1660. 11050560 10.1046/j.1365-294x.2000.01020.x

[ece31673-bib-0010] Crispo, E. , and L. J. Chapman . 2009 Temporal variation in population genetic structure of a Riverine African cichlid fish. J. Hered. 101:97–106. 19734258 10.1093/jhered/esp078

[ece31673-bib-0011] Darriba, D. , G. L. Taboada , R. Doallo , and D. Posada . 2012 jModelTest 2: more models, new heuristics and parallel computing. Nat. Methods 9:772. 22847109 10.1038/nmeth.2109 PMC4594756

[ece31673-bib-0013] Dieringer, D. , and C. Schlötterer . 2003 MICROSATELLITE ANALYZER (MSA): a platform independent analysis tool for large microsatellite data sets. Mol. Ecol. Notes 3:167–169.

[ece31673-bib-0014] Do, C. , R. S. Waples , D. Peel , G. M. Macbeth , B. J. Tillett , and J. R. Ovenden . 2014 NeEstimator v2.0: re‐implementation of software for the estimation of contemporary effective population size (*N* _e_) from genetic data. Mol. Ecol. Resour. 41:209–214. 23992227 10.1111/1755-0998.12157

[ece31673-bib-0015] Drummond, A. J. , M. A. Suchard , D. Xie , and A. Rambaut . 2012 Bayesian phylogenetics with BEAUti and the BEAST 1.7. Mol. Biol. Evol. 29:1969–1973. 22367748 10.1093/molbev/mss075 PMC3408070

[ece31673-bib-0016] Eberhart‐Phillips, L. J. , J. I. Hoffman , E. G. Brede , S. Zefania , M. J. Kamrad , T. Székely , et al. 2015 Contrasting genetic diversity and population structure among three sympatric Madagascan shorebirds: parallels with rarity, endemism, and dispersal. Ecol. Evol. 5:997–1010. 25798218 10.1002/ece3.1393 PMC4364815

[ece31673-bib-0018] Epps, C. W. J. D. , G. K. Roderick , R. B. Ramey , and D. R. McCullough . 2005 Highways block gene flow and cause a rapid decline in genetic diversity of desert bighorn sheep. Ecol. Lett. 8:1029–1038.

[ece31673-bib-2000] ESRI . 2011 ArcGIS Desktop: Release 10. Environmental Systems Research Institute, Redlands, CA.

[ece31673-bib-0019] Evanno, G. , S. Regnaut , and J. Goudet . 2005 Detecting the number of clusters of individuals using the software STRUCTURE: a simulation study. Mol. Ecol. 14:2611–2620. 15969739 10.1111/j.1365-294X.2005.02553.x

[ece31673-bib-0020] Excoffier, L. , and H. L. Lischer . 2010 3.5: a new series of programs to perform population genetics analyses under Linux and Windows. Mol. Ecol. Resour. 10:564–567. 21565059 10.1111/j.1755-0998.2010.02847.x

[ece31673-bib-0021] Faubet, P. , R. S. Waples , and O. E. Gaggiotti . 2007 Evaluating the performance of a multilocus bayesian method for the estimation of migration rates. Mol. Ecol. 16:1149–1166. 17391403 10.1111/j.1365-294X.2007.03218.x

[ece31673-bib-0022] Fernández‐García, J. L. , J. Carranza , J. G. Martínez , and E. Randi . 2014 Mitochondrial D‐loop phylogeny signals two native Iberian red deer (*Cervus elaphus*) Lineages genetically different to Western and Eastern European red deer and infers human‐mediated translocations. Biodiv. Conserv. 23:537–554.

[ece31673-bib-0023] Folmer, O. , M. Black , W. Hoeh , R. Lutz , and R. Vrijenhoek . 1994 DNA primers for amplification of mitochondrial cytochrome c oxidase subunit I from diverse metazoan invertebrates. Mol. Mar. Biol. Biotechnol 3:294–299. 7881515

[ece31673-bib-0024] Fu, Y. X. 1997 Statistical tests of neutrality of mutations against population growth, hitchhiking and background selection. Genetics 147:915–925. 9335623 10.1093/genetics/147.2.915 PMC1208208

[ece31673-bib-0026] Gittenberger, E. 2012 Long‐distance dispersal of molluscs: ‘Their distribution at first perplexed me much’. J. Biogeogr. 39:10–11.

[ece31673-bib-0027] Gittenberger, E. , D. S. J. Groenenberg , B. Kokshoorn , and R. C. Preece . 2006 Biogeography: molecular trails from hitch‐hiking snails. Nature 439:409. 16437103 10.1038/439409a

[ece31673-bib-0028] González‐Astorga, J. , and J. Núñez‐Farfán . 2001 Effect of habitat fragmentation on the genetic structure of the narrow endemic *Brongniartia vazquezii* . Evol. Ecol. Res. 3:861–872.

[ece31673-bib-0029] Green, A. J. , and J. Figuerola . 2005 Recent advances in the study of long‐distance dispersal of aquatic invertebrates via birds. Divers. Distrib. 11:149–156.

[ece31673-bib-0030] Harrison, R. G. 1991 Molecular changes at speciation. Annu. Rev. Ecol. Syst. 22:281–308.

[ece31673-bib-0031] Hurtrez‐Boussès, S. , J. E. Hurtrez , H. Turpin , C. Durand , P. Durand , T. De Meeüs , et al. 2010 Hydrographic network structure and population genetic differentiation in a vector of fasciolosis, *Galba truncatula* . Infect Genet. Evol. 10:178–183. 20085826 10.1016/j.meegid.2010.01.005

[ece31673-bib-0032] Husemann, M. , J. W. Ray , R. S. King , E. A. Hooser , and P. Danley . 2012 Comparative biogeography reveals differences in population genetic structure of five species of stream fishes. Biol. J. Linn. Soc. 107:867–885.

[ece31673-bib-0033] Jakobsson, M. , and N. A. Rosenberg . 2007 CLUMPP: a cluster matching and permutation program for dealing with label switching and multimodality in analysis of population structure. Bioinformatics 23:1801–1806. 17485429 10.1093/bioinformatics/btm233

[ece31673-bib-0035] Kopp, K. C. , K. Wolff , and J. Jokela . 2012 Natural range expansion and human‐assisted introduction leave different genetic signatures in a hermaphroditic freshwater snail. Ecol. Evol. 26:483–498.

[ece31673-bib-0036] Li, X. Y. , Y. Li , S. Q. Zhou , and B. L. Yan . 2010 Analysis and evaluation of nutritional composition in two freshwater fingersnails. Food Sci. 31:276–279.

[ece31673-bib-0037] Li, D. L. , T. Zhang , J. B. Yu , X. W. Mao , H. Q. Wang , K. J. Chen , et al. 2011 Temporal and spatial distribution patterns of mollusca in a typical Aquacultural LAKE—Datong Lake. Acta Hydrobiol. Sin. 35:946–954.

[ece31673-bib-0038] Librado, P. , and J. Rozas . 2009 DnaSP v5: a software for comprehensive analysis of DNA polymorphism data. Bioinformatics 25:1451–1452. 19346325 10.1093/bioinformatics/btp187

[ece31673-bib-0039] Ma, T. , S. Gong , K. Zhou , C. Zhu , K. Deng , Q. Luo , et al. 2010 Laboratory culture of the freshwater benthic gastropod *Bellamya aeruginosa* (Reeve) and its utility as a test species for sediment toxicity. J. Environ. Sci. 22:304–313. 10.1016/s1001-0742(09)60109-1 20397422

[ece31673-bib-0040] Meirmans, P. G. 2014 Nonconvergence in bayesian estimation of migration rates. Mol. Ecol. Res. 14:726–733. 10.1111/1755-0998.12216 24373147

[ece31673-bib-0041] Nalugwa, A. , A. Jørgensen , S. Nyakaana , and T. K. Kristensen . 2011 Genetic variation within and between populations of hermaphroditic *Bulinus truncatus* tetraploid freshwater snails of the Albertine Rift, East Africa. Hydrobiologia 673:53–61.

[ece31673-bib-0043] Nekola, J. C. 2012 The impact of a utility corridor on terrestrial gastropod biodiversity. Biodiv. Conserv. 21:781–795.

[ece31673-bib-0045] Nilsson, C. , C. A. Reidy , M. Dynesius , and C. Revenga . 2005 Fragmentation and flow regulation of the world's large river systems. Science 308:405–408. 15831757 10.1126/science.1107887

[ece31673-bib-0046] Ozawa, K. , A. Yokoyama , K. Ishikawa , M. Kumagai , M. F. Watanabe , and H. D. Park . 2003 Accumulation and depuration of microcystin produced by the cyanobacterium Microcystis in a freshwater snail. Limnology 4:131–138.

[ece31673-bib-0047] Piry, S. , G. Luikart , and J. M. Cornuet . 1999 BOTTLENECK: a computer program for detecting recent reductions in the effective population size using allele frequency data. J. Hered. 90:502–503.

[ece31673-bib-0048] Prabhakar, A. K. , and S. P. Roy . 2009 Ethno‐medicinal uses of some shell fishes by people of Kosi river basin of North‐Bihar, India. Ethno‐Med. 3:1–4.

[ece31673-bib-0049] Pritchard, J. K. , M. Stephens , and P. Donnelly . 2000 Inference of population structure using multilocus genotype data. Genetics 155:945–959. 10835412 10.1093/genetics/155.2.945 PMC1461096

[ece31673-bib-0050] R Development Core Team . 2009 R: a language and environment for statistical computing. R Foundation for Statistical Computing, Vienna, Austria.

[ece31673-bib-1101] Ray, J. W. , M. Husemann , D. J. Lutz‐Carrillo , R. S. King , and P. D. Danley . 2015 Life at the leading edge: genetic impoverishment of the spotted bass, Micropterus punctulatus, at its Western edge. Environmental Biology of Fishes 98:1823‐1832.

[ece31673-bib-0051] Rambaut, A. , and A. J. Drummond . 2009 Tracer 1.5.0. MCMC Trace Analysis Tool. Available at http://beast.bio.ed.ac.uk/Tracer. (accessed 10 April 2014).

[ece31673-bib-1003] Reed, D. H. , and R. Frankham . 2003 Correlation between fitness and genetic diversity. Conservation Biology 17:230‐237.

[ece31673-bib-0052] Ricciardi, A. , and J. B. Rasmussen . 1999 Extinction rates of North American freshwater fauna. Conserv. Biol. 13:1220–1222.

[ece31673-bib-0053] Rice, W. R. 1989 Analyzing tables of statistical tests. Evolution 43:223–225. 10.1111/j.1558-5646.1989.tb04220.x 28568501

[ece31673-bib-0055] Roberts, J. H. , P. L. Angermeier , and E. M. Hallerman . 2013 Distance, dams and drift: what structures populations of an endangered, benthic stream fish? Freshwater Biol. 58:2050–2064.

[ece31673-bib-0056] Roffler, G. H. , S. L. Talbot , G. Luikart , G. K. Sage , K. L. Pilgrim , L. G. Adams , et al. 2014 Lack of sex‐biased dispersal promotes fine‐scale genetic structure in alpine ungulates. Conserv. Genet. 15:837–851.

[ece31673-bib-0057] Rosenberg, N. A. 2004 Distruct: a program for the graphical display of population structure. Mol. Ecol. Notes 4:137–138.

[ece31673-bib-0058] Rousset, F. 2008 Genepop'007: a complete re‐implementation of the Genepop software for Windows and Linux. Mol. Ecol. Res. 8:103–106. 10.1111/j.1471-8286.2007.01931.x 21585727

[ece31673-bib-0059] Schultheiß, R. , T. Wilke , A. Jørgensen , and C. Albrecht . 2011 The birth of an endemic species flock: demographic history of the *Bellamya* group (Gastropoda, Viviparidae) in Lake Malawi. Biol. J. Linn. Soc. 102:130–143.

[ece31673-bib-0061] Sinclair‐Winters, C. M. 2014 Upstream or downstream? Population structure of the land snail *Ventridens ligera* (Say, 1821) in the Potomac River drainage basin. J. Mollus. Stud. 80:280–285.

[ece31673-bib-0062] Song, L. , W. Chen , L. Peng , N. Wan , N. Q. Gan , and X. M. Zhang . 2007 Distribution and bioaccumulation of microcystins in water columns: a systematic investigation into the environmental fate and the risks. associated with microcystins in Meiliang Bay, Lake Taihu. Water Res. 41:2853–2864. 17537477 10.1016/j.watres.2007.02.013

[ece31673-bib-0063] Standley, C. J. , S. L. Goodacre , C. M. Wade , and J. R. Stothard . 2014 The population genetic structure of Biomphalaria choanomphala in Lake Victoria, East Africa: implications for schistosomiasis transmission. Parasite. Vector. 7:524. doi:10.1186/s13071‐014‐0524‐4. 10.1186/s13071-014-0524-4 PMC4254209 25406437

[ece31673-bib-1000] Tang, G. Z. , and M. Tao . 1997 Discussion on relationship between the middle Pleistocene glaciation and formation of the Yangtze Gorges. Geology and Mineral Resources of South China 4:9–18 (in Chinese).

[ece31673-bib-0064] Tajima, F. 1989 Statistical method for testing the neutral mutation hypothesis by DNA polymorphism. Genetics 123:585–595. 2513255 10.1093/genetics/123.3.585 PMC1203831

[ece31673-bib-0065] Thompson, J. D. , T. J. Gibson , F. Plewniak , F. Jeanmougin , and D. G. Higgins . 1997 The Clustal X windows interface: flexible strategies for multiple sequences alignment aided by quality analysis tools. Nucleic Acids Res. 25:4876–4882. 9396791 10.1093/nar/25.24.4876 PMC147148

[ece31673-bib-0066] Tian‐Bi, Y. N. T. , P. Jarne , J. N. K. Konan , J. Utzinger , and E. K. N'Goran . 2013 Contrasting the distribution of phenotypic and molecular variation in the freshwater snail *Biomphalaria pfeifferi*, the intermediate host of *Schistosoma mansoni* . Heredity 110:466–474. 23321708 10.1038/hdy.2012.115 PMC3630815

[ece31673-bib-0067] Tibbets, C. A. , and T. E. Dowling . 1996 Effects of intrinsic and extrinsic factors on population fragmentation in three species of North American minnows (Teleostei: Cyprinidae). Evolution 50:1280–1292. 10.1111/j.1558-5646.1996.tb02368.x 28565290

[ece31673-bib-0068] Toro, M. A. , and A. Caballero . 2005 Characterization and conservation of genetic diversity in subdivided populations. Philos. Trans. R. Soc. Lond. B Biol. Sci. 360:1367–1378. 16048780 10.1098/rstb.2005.1680 PMC1569508

[ece31673-bib-0069] Uit de Weerd, D. R. , D. Schneider , and E. Gittenberger . 2005 The provenance of the Greek land snail species *Isabellaria pharsalica*: molecular evidence of recent passive long‐distance dispersal. J. Biogeogr. 32:1571–1581.

[ece31673-bib-0070] Van Bocxlaer, B. , and G. Hunt . 2013 Morphological stasis in an ongoing radiation from Lake Malawi. Proc. Natl Acad. Sci. USA 110:13892–13897. 23924610 10.1073/pnas.1308588110 PMC3752226

[ece31673-bib-0071] Van Leeuwen, C. H. A. , N. Huig , G. Van der Velde , T. A. Van Alen , C. A. M. Wagemaker , C. D. H. Sherman , et al. 2013 How did this snail get here? Multiple dispersal vectors inferred for an aquatic invasive species. Freshwater Biol. 58:88–99.

[ece31673-bib-0072] Van Oosterhout, C. , W. F. Hutchinson , D. P. M. Wills , and P. Shipley . 2004 MICRO‐CHECKER: software for identifying and correcting genotyping errors in microsatellite data. Mol. Ecol. Notes 4:535–538.

[ece31673-bib-0073] Vellend, M. , and M. A. Geber . 2005 Connections between species diversity and genetic diversity. Ecol. Lett. 8:767–781.

[ece31673-bib-0074] Wada, S. , K. Kawakami , and S. Chiba . 2012 Snails can survive passage through a bird's digestive system. J. Biogeogr. 39:69–73.

[ece31673-bib-0075] Waples, R. S. , and O. Gaggiotti . 2006 What is a population? An empirical evaluation of some genetic methods for identifying the number of gene pools and their degree of connectivity. Mol. Ecol. 15:1419–1439. 16629801 10.1111/j.1365-294X.2006.02890.x

[ece31673-bib-0076] Weir, B. S. , and C. C. Cockerham . 1984 Estimating F‐statistics for the analysis of population structure. Evolution 38:1358–1370. 10.1111/j.1558-5646.1984.tb05657.x 28563791

[ece31673-bib-1004] Willi, Y. , J. Van Buskirk , and A. A. Hoffmann . 2006 Limits to the adaptive potential of small populations. Annual Reviews of Ecology, Evolution and Systematics 37:433‐458.

[ece31673-bib-1005] Wissmann, H. 1937 The Pleistocene glaciation in China. Bulletin of the Geological Society of China 17:145‐168.

[ece31673-bib-0077] Wilke, T. , R. Schultheiß , and C. Albrecht . 2009 As time goes by: a simple fool's guide to molecular clock approaches in invertebrates. Am. Malacol. Bull. 27:25–45.

[ece31673-bib-0078] Wilson, G. A. , and B. Rannala . 2003 Bayesian inference of recent migration rates using multilocus genotypes. Genetics 163:1177–1191. 12663554 10.1093/genetics/163.3.1177 PMC1462502

[ece31673-bib-1006] Xiang, B. X. , Z. Xiang , and Y. H. Xiang . 2006 Investigation of wild Ginkgo biloba in Wuchuan county of Guizhou, China—Guizhou ancient Ginkgo biloba resources investigation VII. Guizhou Sci. 24:56‐67 (in Chinese with an English abstract).

[ece31673-bib-0079] Yang, L. , R. L. Mayden , and S. P. He . 2009 Population genetic structure and geographical differentiation of the Chinese catfish *Hemibagrus macropterus* (Siluriformes, Bagridae): Evidence for altered drainage patterns. Mol. Phylogenet. Evol. 51:405–411. 19405203 10.1016/j.ympev.2009.01.004

[ece31673-bib-0080] Zhang, X. , and Y. Y. Liu . 1960 Morphology and habitat of common *Bellamya* in China. B. Biol. 2:49–57.

[ece31673-bib-0081] Zuberogoitia, I. , H. Zalewskah , J. Zabala , and A. Zalewski . 2013 The impact of river fragmentation on the population persistence of native and alien mink: an ecological trap for the endangered European mink. Biodivers. Conserv. 22:169–186.

