# Supplementary material for: Population genetic structure of Bellamya aeruginosa (Mollusca: Gastropoda: Viviparidae) in China: weak divergence across large geographic distances
Source: Ecol Evol. 2015 Oct 13;5(21):4906–19. doi: 10.1002/ece3.1673 (PMC4662307; doi:10.1002/ece3.1673)
Supplement: Supplementary file 1 — Figure S1. Distributions of pairwise nucleotide differences (mismatch distributions) of COI gene in B. aeruginosa samples and the corresponding Fu's Fs value. Figure S2. The most likely value of K (inferred cluster) estimated using Evanno's ΔK‐method. [file ECE3-5-4906-s001.docx]

Supplemental Figures


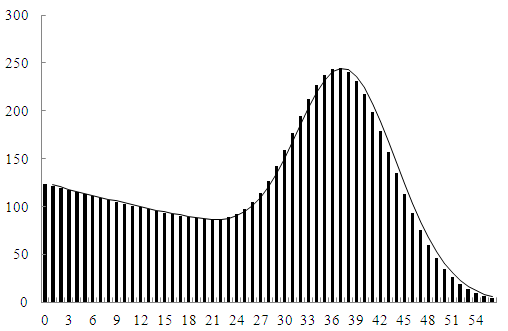


*Fs* =-23.8828（*P* = 0.0008）

Figure S1. Distributions of pairwise nucleotide differences (mismatch distributions) of COI gene in *B. aeruginosa* samples and the corresponding Fu’s *Fs* value. The X-axis represents the number of pairwise differences among sequences and the Y-axis represents the relative frequencies of pairwise comparisons.

Figure S2 The most likely value of *K* (inferred cluster) estimated using Evanno’s ΔK-method
