# Supplementary material for: Population genetic structure of Bellamya aeruginosa (Mollusca: Gastropoda: Viviparidae) in China: weak divergence across large geographic distances
Source: Ecol Evol. 2015 Oct 13;5(21):4906–19. doi: 10.1002/ece3.1673 (PMC4662307; doi:10.1002/ece3.1673)
Supplement: Supplementary file 2 — Table S1. Primer sequence for microsatellite markers and optional PCR conditions. Table S2. The frequencies of null alleles across seven microsatellite loci and 12 geographic locations for B. aeruginosa, as suggested by the program MICRO‐CHECKER. [file ECE3-5-4906-s002.doc]

Table S1. Primer sequence for microsatellite markers and optional PCR conditions

| Locus | Repeated motif | Primer (5' to 3') | Fluorescent | Tm ℃ |
| --- | --- | --- | --- | --- |
| ATG142 | (ATG)_n_ | ATGCCGATTATTCTGATTCTGG | 5'-HEX | 53 |
|  |  | ACGCAAGTTTCATTCATGTATGTC |  |  |
| ATG146 | (ATG)_n_ | GCTCTGTCCAGCAAGAAACTAG | 5'-TAMRA | 55 |
|  |  | ATAGACATCAGTCCGACAAAGC |  |  |
| TC298 | (AG)_n_ | CTCCAAAGACTGTTACTGCTACGA | 5'-HEX | 57 |
|  |  | CACACAAACTAGGTAAGGGGACAT |  |  |
| GATA328 | (TCTA)_n_ | CCTGCGTCAATTTAAAACCATAG | 5'-FAM | 55 |
|  |  | GGGTAGGTAGGTGGGTAAGTGAG |  |  |
| CAG41 | (TGC)_n_ | TTTGCTGCGTTTACTCGTCCTG | 5'-HEX | 55 |
|  |  | CTCGTTCTTGGGCTGGGTGTT |  |  |
| CAG178 | (CAG)_n_ | CATGACATAACACCCCTACCCTT | 5'-TAMRA | 55 |
|  |  | AATTTGGTATCTTGGAATCTGACG |  |  |
| CCT238 | (GT)_n_ | ACATAAGTGCTTGCGATAGTGCG | 5'-TAMRA | 57 |
|  |  | ATCCCAGTGTCCTCAACCTAAACG |  |  |

Table S2. The frequencies of null alleles across seven microsatellite loci and 12 geographic locations for *B. aeruginosa*, as suggested by the program MICRO-CHECKER

| Locus | CH | DT | PY | QJ | HZ | NS | BY | YX | LZ | HH | EH | DC |
| --- | --- | --- | --- | --- | --- | --- | --- | --- | --- | --- | --- | --- |
| ATG142 |  |  |  | 0.149 | 0.119 |  | 0.127 | 0.197 |  |  |  |  |
| ATG146 | 0.073 | 0.173 | 0.077 | 0.120 |  | 0.097 |  | 0.084 | 0.107 |  |  |  |
| CAG41 |  | 0.186 | 0.239 | 0.244 |  | 0.104 | 0.185 |  |  |  |  | 0.160 |
| CAG178 | 0.171 |  |  | 0.180 | 0.186 | 0.189 |  |  |  |  |  |  |
| CCT238 | 0.109 | 0.100 | 0.130 |  |  |  |  |  |  |  |  |  |
| GATA328 |  |  |  |  |  |  |  |  |  |  |  |  |
| TC298 |  |  |  |  | 0.121 |  | 0.103 |  |  | 0.104 | 0.093 | 0.118 |
